# Early initiation of continuous renal replacement therapy improves survival of elderly patients with acute kidney injury: a multicenter prospective cohort study

**DOI:** 10.1186/s13054-016-1437-8

**Published:** 2016-08-16

**Authors:** Jae Yoon Park, Jung Nam An, Jong Hyun Jhee, Dong Ki Kim, Hyung Jung Oh, Sejoong Kim, Kwon Wook Joo, Yun Kyu Oh, Chun-Soo Lim, Shin-Wook Kang, Yon Su Kim, Jung Tak Park, Jung Pyo Lee

**Affiliations:** 1 Department of Internal Medicine, Dongguk University Ilsan Hospital, 27 Dongguk-ro, Ilsandong-gu, Goyang-si, Gyeonggido 10326 Korea; 2 Department of Internal Medicine, Seoul National University College of Medicine, 28 Yongon-dong, Chongno-gu, Seoul 110-744 Korea; 3 Department of Critical Care Medicine, Seoul National University Boramae Medical Center, 20 Boramae-ro 5-gil, Dongjak-gu, Seoul 07061 Korea; 4 Department of Internal Medicine, College of Medicine, Institute of Kidney Disease Research, Yonsei University, 50-1 Yonsei-ro, Seodaemun-gu, Seoul 03722 Korea; 5 Department of Internal Medicine, Seoul National University Bundang Hospital, 82 Gumi-ro 173beon-gil, Bundang-gu, Seongnam-si, Gyeonggi-do 13620 Korea; 6 Department of Internal Medicine, Seoul National University Boramae Medical Center, 20 Boramae-ro 5-gil, Dongjak-gu, Seoul 07061 Korea

**Keywords:** Elderly patients, Survival, Continuous renal replacement therapy, Acute kidney injury, Propensity score matching

## Abstract

**Background:**

Continuous renal replacement therapy (CRRT) is essential in the management of critically ill patients with acute kidney injury (AKI). However, the optimal timing for initiating CRRT remains controversial, especially in elderly patients. Therefore, we investigated the outcomes of early CRRT initiation in elderly patients with AKI.

**Methods:**

A total of 607 patients ≥65 years of age who started CRRT due to AKI between August 2009 and December 2013 were prospectively enrolled. They were divided into two groups based on the median 6-hour urine output immediately before CRRT initiation. Propensity score matching was used to compare the overall survival rate, CRRT duration, and hospitalization duration.

**Results:**

The median age of both groups was 73.0 years, and 60 % of the patients were male. The most common cause of AKI was sepsis. In the early CRRT group, the mean arterial pressure was higher, but the prothrombin time and total bilirubin, aspartate aminotransferase, and alanine aminotransferase levels were lower. The overall cumulative survival rate was higher in the early CRRT group (log-rank *P* < 0.01). Late CRRT initiation was associated with a higher mortality rate than early initiation after adjusting for age, sex, the Charlson comorbidity index, systolic arterial pressure, prothrombin time, the total bilirubin, aspartate aminotransferase, and alanine aminotransferase levels, cumulative fluid balance and diuretic use (hazard ratio, 1.35; 95 % confidence interval 1.06, 1.71, *P* = 0.02). Following propensity score matching, patient survival was significantly better in the early CRRT group than in the late CRRT group (*P* < 0.01). The total duration of hospitalization from the start of CRRT was shorter among the survivors when CRRT was started earlier (26.7 versus 39.1 days, *P* = 0.04).

**Conclusion:**

A better prognosis can be expected if CRRT is applied early in the course of AKI in critically ill, elderly patients.

**Electronic supplementary material:**

The online version of this article (doi:10.1186/s13054-016-1437-8) contains supplementary material, which is available to authorized users.

## Background

Elderly people (aged ≥65 years) are currently the fastest-growing sector of the general population in developed countries. They are more prone to developing acute kidney injury (AKI) because of structural and functional alterations in the kidney [1, 2], comorbidities (e.g., arteriosclerosis, hypertension, diabetes mellitus, and heart failure), and polypharmacy for the treatment of comorbidities, which increase in prevalence with age. Accordingly, an increasing number of elderly patients can be expected to develop AKI [3–5].

For more than a decade, continuous renal replacement therapy (CRRT) has been essential in the management of critically ill patients with AKI [6, 7]. The generally accepted indications for initiating CRRT in AKI include persistent hyperkalemia, severe acidosis and hypervolemia that are unresponsive to adequate medical management, and overt uremic symptoms or signs (e.g., uremic bleeding, pericarditis, and encephalopathy) [8–10].

Although the optimal timing of CRRT initiation remains controversial, recent studies comparing early and late initiation have suggested that early initiation may have a beneficial impact on survival [11–14]. However, elderly patients are more susceptible to hemodynamic complications during dialysis, such as intradialytic hypotension and arrhythmia, because of decreased autonomic function and cardiovascular reserve, bleeding problems, and neurological complications resulting from rapid changes in serum electrolytes and osmolarity [15, 16]. Additionally, because short-term and long-term survival following CRRT are expected to be lower in elderly patients compared with the general population [17], therapeutic decisions, including use of CRRT, tend to be more conservative for patients in this age group, especially in intensive care. Nevertheless, few studies have specifically examined CRRT in older individuals with AKI. Therefore, although previous studies have demonstrated that early initiation of CRRT could be beneficial in the general population, it is not clear whether it could also benefit elderly patients.

In the present study, we aimed to investigate the outcomes of early versus late initiation of CRRT using propensity score matching (PSM) in a multicenter, prospective CRRT cohort of elderly individuals.

## Methods

### Study population

All patients aged ≥18 years who received CRRT for AKI at Seoul National University Hospital, Seoul National University Boramae Hospital, and Yonsei University Severance Hospital were initially screened (n = 1471). The patients were prospectively enrolled between August 2009 and December 2013. We excluded 724 patients who were younger than 65 years of age and 140 who were on chronic dialysis. A total of 607 patients were included in the analyses. They were divided into two groups based on the body-weight-adjusted median 6-h urine output immediately before administration of CRRT.

### Definitions

The definition of AKI was based on the Kidney Disease: Improving Global Outcomes (KDIGO) clinical practice guidelines for AKI and was defined as the presence of at least one of the following criteria: an increase in the serum creatinine level ≥0.3 mg/dL (≥26.5 μmol/L) within 48 h; an increase in the serum creatinine level to ≥1.5 times the baseline level that was known or was presumed to have occurred within the previous 7 days; or urine volume <0.5 mL/kg/h for 6 h. For the diagnosis of systemic inflammatory response syndrome, the presence of at least two of the following criteria were required: core temperature ≥38 °C or ≤36 °C; heart rate ≥90 beats per minute; respiratory rate ≥20 breaths per minute and P_CO2_ ≤ 32 mmHg or mechanical ventilation; and peripheral leukocyte count ≥12,000/mm^3^ or ≤4000/mm^3^ [18]. Septic AKI was defined as systemic inflammatory response syndrome combined with an infectious episode and AKI.

### Clinical outcomes

The primary outcome was the patient survival rate after CRRT initiation. The secondary outcomes were mortality on the 28^th^ day of CRRT, the durations of CRRT and total renal replacement therapy (RRT), and the durations of total hospitalization and stay in the intensive care unit (ICU) from CRRT initiation.

### Data collection

At the time of CRRT initiation, demographic, clinical, and laboratory data were collected. For the assessment of disease severity, the Charlson comorbidity index (CCI), sequential organ failure assessment (SOFA) score, and acute physiology and chronic health evaluation (APACHE) II score were calculated at the time of dialysis initiation [19, 20]. All available intake and output data for 3 days immediately before CRRT initiation were used to calculate the cumulative fluid balance. The estimated glomerular filtration rate (eGFR) was also calculated using the modification of diet in renal disease equation [21]. The patients were stratified into two groups (patients with early or late CRRT) based on the body-weight-adjusted median 6-h urine output before CRRT initiation.

### Statistical analysis

Categorical variables described as frequencies and proportions were compared using the chi-squared test. Following a test for normality, the non-normally distributed variables were expressed as the median (25–75^th^ percentiles) and were compared using the Mann–Whitney *U* or Kruskal-Wallis test. The normally distributed variables were presented as the mean ± standard deviation. Patient survival was estimated by Kaplan-Meier curves and multivariate Cox regression models based on the body-weight-adjusted median 6-h urine output immediately before CRRT initiation. Propensity scores were estimated by multiple logistic regression analysis with adjustments for patient age, sex, the CCI, systolic arterial pressure, prothrombin time, and the total bilirubin level. After calculation of the propensity scores, we matched patients in the early and late CRRT groups with similar propensity scores at a 1:1 ratio using the nearest neighbor method, no replacement, and a 0.2 caliper width. PSM was used to increase the precision of the estimated effect without increasing bias due to the presence of variables potentially associated with survival [22]. The characteristics of both the early and late CRRT groups were compared before and after PSM. Kaplan-Meier survival curves and life tables were generated for the two groups after PSM.

All statistical tests were evaluated using a two-tailed 95 % confidence interval (CI), and a *P* value <0.05 was considered statistically significant. All descriptive and survival analyses were performed using SPSS for Windows, version 21.0 (IBM, Armonk, NY, USA). R software (version 2.14.2) was used for PSM.

## Results

### Baseline characteristics of the subjects

The baseline characteristics of the 607 patients are listed in Table 1. At CRRT initiation, the median patient age was 73.0 years, and 60.1 % of the patients were male.

**Table 1:** Baseline characteristics of the two groups stratified by median pre-CRRT 6-h urine output before and after propensity score matching at the time of CRRT initiation

	Before propensity score matching				After propensity score matching			
Variable	Early CRRT (N = 303)	Late CRRT (N = 304)	*P* value	SD	Early CRRT (N = 241)	Late CRRT (N = 241)	*P* value	SD
Age (years)	75.1 (69.7–80.0)	74.0 (70.0–80.0)	0.80	−0.02	76.0 (70.2–80.6)	73.5 (69.5–80.0)	0.5	0.04
Sex, male (*n* (%))	180 (59.4 %)	185 (60.9 %)	0.74	−0.01	145 (60.2 %)	145 (60.2 %)	1.00	0.03
Body mass index (kg/m^2^)	23.1 (20.1–25.8)	22.5 (20.0–25.0)	0.64		23.0 (20.2–25.6)	22.2 (19.7–25.0)	0.61	
Cause of acute kidney injury (*n* (%))			0.37				0.28	
Septic	138 (45.5 %)	137 (45.1 %)			116 (48.1 %)	116 (48.1 %)		
Nephrotoxic	27 (8.9 %)	18 (5.9 %)			20 (8.3 %)	12 (5.0 %)		
Ischemic	49 (16.2 %)	63 (20.7 %)			37 (15.4 %)	46 (19.1 %)		
Postoperative	34 (11.2 %)	25 (8.2 %)			26 (10.8 %)	21 (8.7 %)		
Others	55 (18.2 %)	61 (20.1 %)			42 (17.4 %)	46 (19.1 %)		
Time from ICU admission to CRRT initiation (h)	6.8 (1.5–31.1)	7.9 (1.7–37.8)	0.30		7.0 (1.6–32.0)	7.7 (1.6–32.1)	0.95	
Charlson comorbidity index	7.0 (4.0–11.0)	5.0 (4.0–8.0)	0.23	−0.05	7.0 (4.0–11.0)	5.0 (4.0–8.0)	0.70	0.02
SOFA score	11.6 ± 3.7	12.5 ± 3.4	0.08		11.8 ± 3.6	12.4 ± 3.4	0.11	
APACHE II score	28.5 ± 7.4	29.6 ± 7.1	0.19		29.2 ± 7.4	29.5 ± 7.1	0.65	
Mechanical ventilation needs (*n* (%))	251 (82.8 %)	253 (83.5 %)	0.78		203 (84.2 %)	200 (83.0 %)	0.60	
FiO_2_	0.5 (0.4–0.5)	0.5 (0.4–0.5)	0.76		0.5 (0.4–0.5)	0.5 (0.4–0.5)	0.71	
Systolic blood pressure (mmHg)	115.1 ± 24.7	109.3 ± 24.8	<0.01	0.31	111.9 ± 22.0	109.7 ± 24.7	0.30	0.08
Diastolic blood pressure (mmHg)	61.8 ± 14.0	61.0 ± 15.0	0.48		61.2 ± 13.1	61.8 ± 15.1	0.63	
Mean arterial pressure (mmHg)	79.6 ± 15.6	77.1 ± 16.3	0.05		78.1 ± 14.1	77.8 ± 16.3	0.82	
Six-hour urine volume before CRRT initiation (mL)	230.0 (150.0–432.0)	20.0 (0.0–40.0)	<0.01		220.0 (147.5–420.0)	23.0 (0.0–43.8)	<0.01	
Cumulative fluid balance (mL)	1951.0 (424.8–3435.5)	2025.0 (0.0–4520.0)	0.06		1961.0 (544.5–3635.5)	2072.7 (0.0–4655.0)	0.37	
Diuretics use (*n* (%))	209 (69.0 %)	194 (63.8 %)	0.20		164 (68.0 %)	148 (61.4 %)	0.15	
Target clearance (mL/kg/h)	40.0 (38.1–50.0)	40.0 (36.7–47.1)	0.88		40.0 (38.4–49.5)	40.0 (37.2–46.6)	0.70	
Dialysate flow rate (mL/h)	1250.0 (1000.0–1550.0)	1100.0 (1000.0–1500.0)	0.23		1200.0 (1000.0–1500.0)	1100.0 (1000.0–1475.0)	0.34	
Replacement flow rate (mL/h)	1200.0 (1000.0–1500.0)	1100.0 (1000.0–1500.0)	0.13		1200.0 (1000.0–1500.0)	1100.0 (1000.0–1475.0)	0.17	
Blood flow rate (mL/min)	100.0 (100.0–100.0)	100.0 (100.0–100.0)	0.70		100.0 (100.0–100.0)	100.0 (100.0–100.0)	0.38	
Biochemical data								
White blood cells (*n*/μL)	12370.0 (7600.0–20500.0)	12975.0 (8495.0–24542.5)	0.11		12370.0 (7370.0–20570.0)	12975.0 (8500.0–24522.5)	0.06	
Hemoglobin (g/dL)	9.7 (8.6–11.3)	9.4 (8.0–11.2)	0.38		9.8 (8.6–11.4)	9.4 (8.0–11.4)	0.76	
Platelets (×10^3^/μL)	104.0 (59.0–161.0)	96.0 (60.0–167.5)	0.42		98.0 (57.5–159.0)	96.0 (60.0–169.0)	0.68	
PT-INR	1.4 (1.2–1.9)	1.7 (1.4–2.6)	<0.01	−0.74	1.5 (1.2–2.0)	1.7 (1.3–2.4)	0.16	−0.22
Albumin (g/dL)	2.7 ± 0.5	2.7 ± 0.6	0.96		2.6 ± 0.5	2.7 ± 0.6	0.35	
Total bilirubin (mg/dL)	1.1 (0.8–.0)	1.6 (0.8–3.9)	<0.01	−0.43	1.2 (0.8–2.2)	1.5 (0.8–3.3)	0.18	−0.08
Aspartate aminotransferase (IU/L)	68.0 (36.0–212.0)	198.5 (52.0–1049.8)	<0.01		73.0 (36.0–254.5)	168.0 (47.3–1020.8)	<0.01	
Alanine aminotransferase (IU/L)	27.0 (16.0–117.0)	82.0 (19.0–379.0)	<0.01		28.0 (16.0–126.5)	75.5 (19.0–347.5)	<0.01	
Blood urea nitrogen (mg/dL)	49.0 (35.0–71.0)	43.0 (32.0–65.5)	0.61		49.0 (33.0–69.0)	43.5 (32.0–63.5)	0.54	
Creatinine (mg/dL)	2.8 (2.0–3.7)	2.7 (2.0–4.0)	0.07		2.7 (2.0–3.4)	2.7 (2.0–4.0)	0.01	

The data are presented as the median (25-75^th^ percentiles), mean ± standard deviation, or as number (percent). *SD* standardized difference, *CRRT* continuous renal replacement therapy, *ICU* intensive care unit, *SOFA* sequential organ failure assessment, *APACHE II* acute physiology and chronic health evaluation II, *FiO*_*2*_ inspired oxygen fraction, *PT-INR* prothrombin time-international normalized ratio, *eGFR* estimated glomerular filtration rate

Before PSM, 303 patients were in the early CRRT group, and 304 were in the late CRRT group. Sepsis (45.3 %) was the most common cause of AKI, followed by ischemia (18.5 %) and postoperative (9.7 %) and nephrotoxic (7.4 %) causes. The median 6-h urine output before CRRT initiation was 0.24 mL/kg/h. The patients were divided into two groups based on the median 6-hour urine output: early (≥0.24 mL/kg/h) and late (<0.24 mL/kg/h) CRRT groups. The 6-hour urine volumes before CRRT initiation were 230.0 (150.0–432.0) mL in the early CRRT group and 20.0 (0.0–40.0) mL in the late CRRT group. There was no difference in cumulative fluid balance or diuretic use between the two groups. The time from ICU admission to CRRT initiation was 6.8 (1.5–31.1) h in the early CRRT group and 7.9 (1.7–37.8) h in the late CRRT group (*P* = 0.30). The CCI, SOFA score, and APACHE II score did not differ between the two groups. The number of patients who required mechanical ventilation and the fraction of inspired oxygen (0.5 (0.4-0.5) versus 0.5 (0.4–0.5), *P* = 0.76) also did not differ between the two groups. The systolic blood pressure (SBP) and mean arterial pressure were higher in the early CRRT group. However, the target clearance and initial blood flow rate for CRRT did not differ between the groups. The serum creatinine level, eGFR, white blood cell count, hemoglobin concentration, and platelet count also did not differ between the groups. The prothrombin time-international normalized ratio (PT-INR) and serum total bilirubin, aspartate aminotransferase (AST), and alanine aminotransferase (ALT) levels were higher in the late CRRT group.

All of the patients in the two groups were matched by propensity scores for the timing of initiation of CRRT using the following covariates: age, sex, the CCI, SBP, the PT-INR, and the total bilirubin level. After PSM, 482 patients (241 in each group) remained. The distributions of the propensity scores before and after matching are shown in the supplementary data section (Additional file 1: Figure S1). Almost all of the baseline parameters, including age, sex, body mass index (BMI), the cause of AKI, time from ICU admission to CRRT initiation, the CCI, SOFA score, APACHE II score, mechanical ventilation requirements, arterial pressures, CRRT setting, and biochemical data, except for the AST, ALT, and creatinine levels, were similar between the propensity score-matched patients in the early and late CRRT groups. In addition, the propensity scores of the matched patients did not differ between the groups.

### Survival analyses stratified by timing of CRRT initiation

**Fig. 1:**
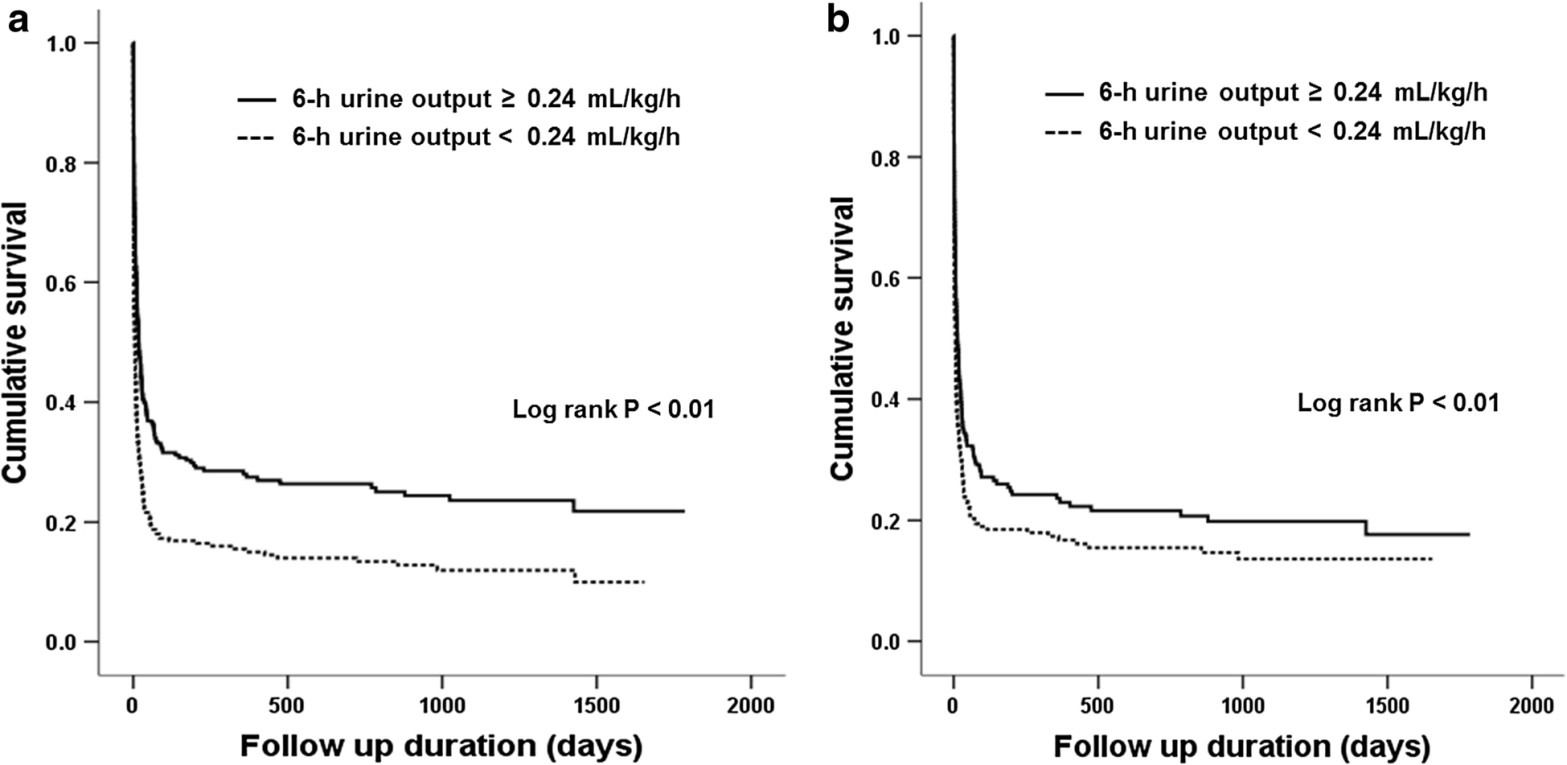
Survival curves obtained using the Kaplan-Meier method for the cohort differentiated by the median 6-h urine output before (**a**) and after (**b**) propensity score matching

**Fig. 2:**
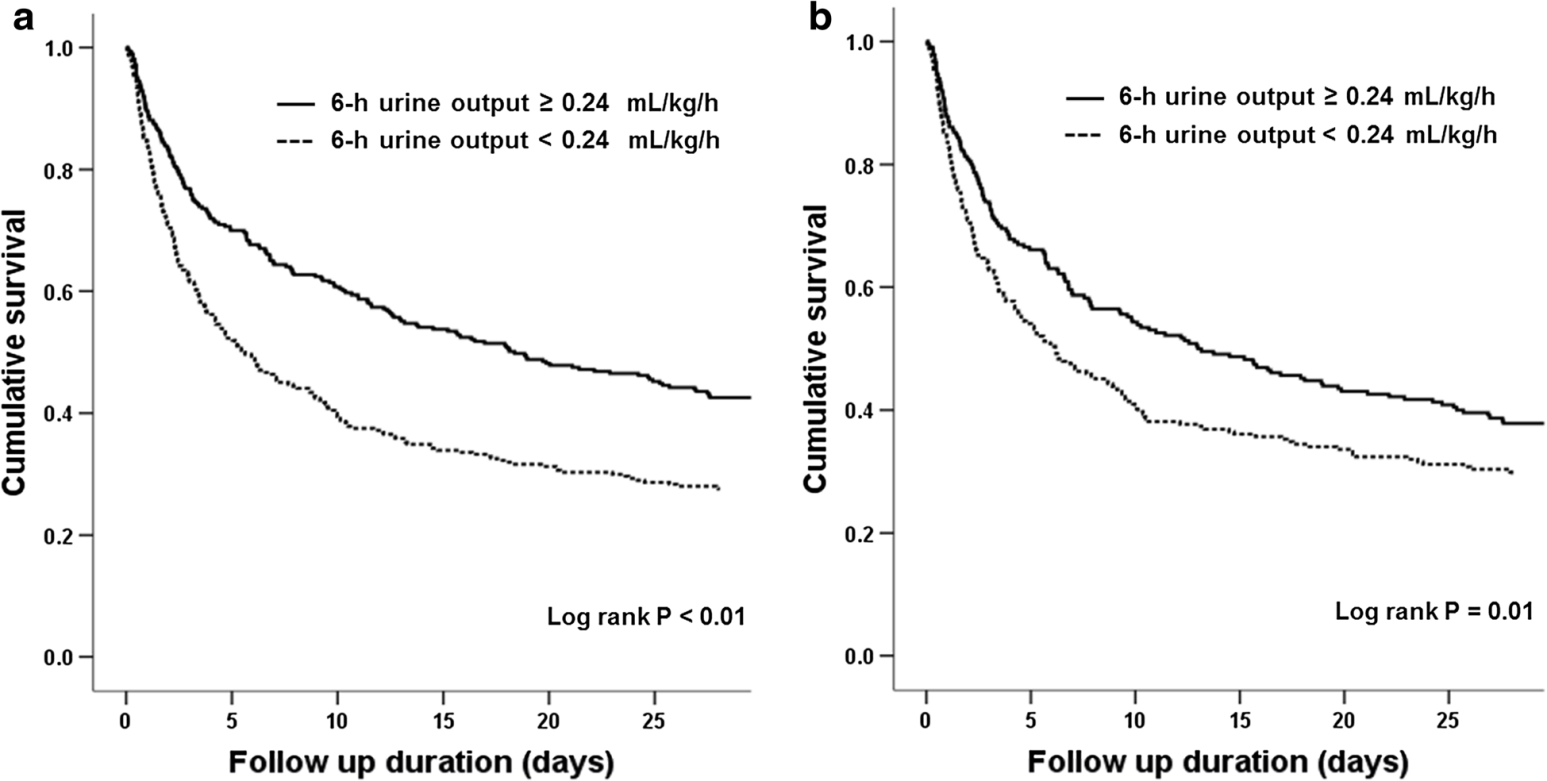
Kaplan-Meier plots of cumulative 28-day survival stratified by the median 6-h urine output before (**a**) and after (**b**) propensity score matching

There were 490 mortalities (79.2 %) during the median follow up of 9.6 days. Figure 1 shows the survival curves obtained using the Kaplan-Meier method; these curves were differentiated according to the body-weight-adjusted median 6-h urine output. The early CRRT group had a significantly higher cumulative survival rate than the late CRRT group (*P* < 0.01), and increased survival after PSM (*P* < 0.01). Univariate and multivariate Cox regression analyses were also performed (Table 2). Before PSM, in an unadjusted model the late CRRT group had an increased hazard ratio (HR) for mortality (model 1: HR 1.60, 95 % CI 1.33, 1.92, *P* < 0.01). This association was significant after adjusting for age, sex, and the CCI (model 2: HR 1.60, 95 % CI 1.33, 1.91, *P* < 0.01) or for SBP, the PT-INR, total bilirubin, AST, and ALT levels, cumulative fluid balance and use of diuretics (model 3: HR 1.35, 95 % CI 1.06, 1.71, *P* = 0.02). Furthermore, the association remained significant after PSM (model 4: HR 1.32, 95 % CI 1.08, 1.61, *P* < 0.01). The 28-day cumulative survival rate was higher in the early CRRT group (Fig. 2, *P* < 0.01), and this trend also remained significant after PSM (*P* = 0.01).

**Table 2:** Multivariate analysis of risk factors for mortality in elderly patients with severe acute kidney injury in the group with late initiation of continuous renal replacement therapy (CRRT) compared to the group with early initiation of CRRT group, using Cox regression models and a propensity score-matched model

	Hazard ratio	95 % Confidence interval	*P* value
Model 1^a^	1.60	1.33-1.92	<0.01
Model 2^b^	1.60	1.33-1.91	<0.01
Model 3^c^	1.35	1.06-1.71	0.02
Model 4^d^	1.32	1.08-1.61	<0.01

^a^Unadjusted. ^b^Adjusted for age, sex, and the Charlson comorbidity index. ^c^Adjusted for age, sex, the Charlson comorbidity index, systolic blood pressure, prothrombin time, total bilirubin, aspartate aminotransferase, alanine aminotransferase, cumulative fluid balance and use of diuretics. ^d^Propensity score-matched; covariates for matching: age, sex, the Charlson comorbidity index, systolic blood pressure, prothrombin time, and total bilirubin

### Duration of RRT and hospitalization

Before PSM, the durations of CRRT were 3.0 (0.9–6.1) days in the early CRRT group and 2.4 (1.0–5.2) days in the late CRRT group (*P* = 0.10) (Table 3). Although the durations of total RRT (3.6 (1.1–10.7) versus 3.1 (1.1–9.6) days, *P* = 0.02) and ICU stay from CRRT initiation (6.2 [1.8-11.4] versus 4.1 [1.4-9.3] days, *P* < 0.01) were longer in the early CRRT group, there was no significant differences in the duration of CRRT, total RRT, or ICU stay among the survivors. While the duration of hospitalization from CRRT initiation was longer in the early CRRT group (16.5 (3.8–37.5) versus 6.1 (1.4–29.6) days, *P* < 0.01), it was shorter among the survivors in this group (25.7 (16.5–38.6) versus 38.8 (27.2–79.3), *P* < 0.01).

**Table 3:** Comparison of duration of dialysis and hospital stay before and after propensity score matching

	Before propensity score matching						After propensity score matching					
	All patients			Survivors			All patients			Survivors		
Characteristic	Early CRRT (N = 303)	Late CRRT (N = 304)	*P* value	Early CRRT (N = 75)	Late CRRT (N = 41)	*P* value	Early CRRT (N = 241)	Late CRRT (N = 241)	*P* value	Early CRRT (N = 59)	Late CRRT (N = 40)	*P* value
Duration of CRRT (days)	3.0 (0.9–6.1)	2.4 (1.0–5.2)	0.10	3.2 (2.1–6.3)	3.8 (2.4–6.9)	0.16	3.0 (0.9–6.6)	2.4 (0.8–5.1)	0.12	3.5 (2.5–6.8)	3.9 (2.4–6.9)	0.47
Duration of total RRT (days)	3.6 (1.1–10.7)	3.1 (1.1–9.6)	0.02	5.9 (2.3–12.9)	6.7 (3.7–27.4)	0.08	3.4 (1.0–10.2)	2.9 (1.0–9.4)	0.16	5.9 (3.0–11.3)	6.7 (4.2–27.4)	0.08
Duration of ICU stay from CRRT initiation (days)	6.2 (1.8–11.4)	4.1 (1.4–9.3)	<0.01	9.0 (4.8–19.8)	9.0 (5.0–19.9)	0.72	6.2 (1.7–11.0)	3.9 (1.3–9.1)	0.02	9.3 (4.9–18.5)	9.4 (5.5–19.9)	0.99
Total duration of hospitalization from CRRT initiation (days)	16.5 (3.8–37.5)	6.1 (1.4–29.6)	<0.01	25.7 (16.5–38.6)	38.8 (27.2–79.3)	<0.01	15.5 (3.4–36.6)	6.1 (1.3–36.7)	0.08	26.7 (16.1–38.2)	39.1 (27.7–79.3)	0.04

The data are presented as the median (25-75^th^ percentiles). *CRRT* continuous renal replacement therapy, *RRT* renal replacement therapy, *ICU* intensive care unit

After PSM, there was no significant difference in the duration of CRRT or total RRT between all of the patients and the survivors. Although the duration of ICU stay from CRRT initiation was longer in the early CRRT group (6.2 (1.7–11.0) versus 3.9 (1.3–9.1), *P* = 0.02), among the survivors there was no difference between the groups. Further, while the total duration of hospitalization did not differ between the groups, it was shorter among the survivors in the early CRRT group (26.7 (16.1–38.2) versus 39.1 (27.7–79.3) days, *P* = 0.04).

## Discussion

In the present study, we compared the outcomes of CRRT among elderly patients according to the timing of CRRT initiation, with stratification by the median 6-h urine output immediately before initiation, using prospective data from 607 patients aged ≥65 years who started CRRT due to AKI between 2009 and 2013 at three centers. The results indicated a survival benefit of early CRRT initiation.

The question of when to initiate dialysis in patients with AKI has been debated almost as long as hemodialysis has been an integral part of the treatment of patients with AKI in clinical medicine. In general, CRRT has been indicated for patients with severe AKI presenting with intractable metabolic derangement or uremic complications. Additionally, many factors must be considered in deciding when to initiate CRRT, including the patient’s demographic data, socio-economic status, and clinical circumstances [8–10, 23]. Therefore, the optimal timing of CRRT initiation has been difficult to investigate and varies substantially among clinical practitioners.

“Early” or “prophylactic” RRT was first introduced in the 1950s. Early literature published in the 1960s and 1970s suggests that early initiation of RRT may improve survival [24–30]. These studies defined early initiation of dialysis as blood urea nitrogen (BUN) ranging from approximately <100 mg/dL to 150 mg/dL and late initiation as BUN ranging from approximately 160 mg/dL to >200 mg/dL. However, those studies had poor experimental design, including inadequate, small sample sizes and the use of arbitrary definitions for early initiation of RRT. In one randomized controlled trial (RCT) that analyzed the outcomes of 34 patients with AKI in the 1980s, dialysis was performed to maintain predialysis BUN at <60 mg/dL in an intensive treatment arm and at 100 mg/dL in a non-intensive arm [31]. Although survival was lower in the intensively treated group, this difference was not significant because of the small sample size.

In the late 1990s and early 2000s, several studies evaluating the impact of the timing of CRRT initiation also addressed the potential survival benefit of early dialysis. Survival was better with early CRRT initiation (39 % versus 20 %) in a study conducted in 1999, involving retrospective analysis of 100 patients who received CRRT to treat post-traumatic AKI [32]. However, in this study the definition of the timing of initiation of dialysis was arbitrary and multivariate analysis was not performed to correct for possible confounders, such as age and serum albumin level. Similar data were reported in the 2000s in three retrospective analyses of the timing of CRRT initiation in patients following cardiac surgery [33, 34] and in oliguric patients with sepsis [35]. In the two former post-cardiac-surgery studies, oliguria, defined as urine volume of <100 mL over 8 h despite furosemide administration, was used as an initiation criterion for early CRRT and azotemia (serum creatinine >5 mg/dL [33] or BUN ≥84 mg/dL and serum creatinine ≥2.8 mg/dL [34]) and hyperkalemia (serum potassium >5.5 mEq/L [33] or >6.0 mEq/L [34]) as a criterion for late CRRT. Both studies demonstrated better survival in the early dialysis group. In the latter study of patients with oliguria and sepsis, there were also more favorable survival outcomes in the group that with early initiation of CRRT. Similar to many previous studies, BUN was used in this study as a surrogate marker for the extent of AKI. However, BUN is suboptimal for estimating renal function because it is affected not only by glomerular filtration but also by renal tubular control, protein intake, protein catabolism, various clinical conditions (e.g., gastrointestinal bleeding), and pharmacological therapy (e.g., corticosteroids) [36].

The timing of RRT in critically ill patients with AKI is still the subject of controversy despite recent RCTs. Two RCTs evaluating the effects of early versus late initiation of RRT in critically ill patients with AKI have been published in 2016 [37, 38]. One single-center RCT from Germany included 231 critically ill patients with KDIGO stage 2 AKI and plasma neutrophil gelatinase-associated lipocalin (NGAL) >150 ng/Ml, and defined early RRT as initiation within 8 h of diagnosis of stage 2 AKI and delayed RRT as initiation within 12 h of stage 3 AKI [37]. The researchers reported that early RRT initiation reduced mortality during the first 90 days.

Another multicenter RCT from France included 619 patients with severe AKI classified as KDIGO stage 3, who required mechanical ventilation, catecholamine infusion, or both, and did not have a potentially life-threatening complication directly related to renal failure [38]. The researchers defined early RRT as initiation immediately after randomization and late RRT as initiation in patients with at least one of the following criteria: severe hyperkalemia, metabolic acidosis, pulmonary edema, BUN > 112 mg/dL, and/or oliguria for more than 72 h after randomization. They concluded that there was no significant difference in 60-day mortality between the early and late groups.

In the present study, we defined the elderly population as individuals ≥65 years of age. Global life expectancy at any age has increased, and the size of the population above 65 years of age is rapidly increasing in developed countries [39]. Along with the increasing size of the elderly population, the incidence of AKI is also rising [40–42]. Because of structural and functional changes in the kidneys, the presence of multiple comorbidities, and the resulting medication use, the elderly population is less able to adapt to rapid hemodynamic changes and alterations in electrolyte levels and osmolality [1–5]. Moreover, as with other life-sustaining therapies, economic and ethical concerns must be considered in making decisions on the initiation of RRT in the elderly population [43, 44].

According to previous studies involving intermittent hemodialysis, elderly patients with AKI generally tolerate hemodialysis well, despite their increased fragility [45]. Further, a critical care study has demonstrated that although elderly individuals have increased mortality risk, intensive care in this population is generally cost-effective [44]. Although several previous studies have reported a survival benefit of early CRRT in relatively older patients with AKI [33, 46, 47], it does not seem appropriate to use these data to predict the outcomes of elderly patients. In 2004, in two studies that included 64 and 61 AKI patients who required CRRT after cardiac surgery respectively [33, 46], the conclusions was that early CRRT initiation is beneficial. However, the ages of the patients in these studies were younger than in our study. In particular, the study performed in Turkey included over 80 % of patients younger than 70 years of age [46], while 74 % of the participants in our study were over 70 years of age, and the youngest patient was 65. Further, a study published in 2013 included patients with a mean age of 61 years. However, the standard deviation was relatively large, indicating that most of the patients were under 65 years of age [47]. In addition, that study reported short-term outcome (28-day survival) only. In contrast, we presented both short-term and long-term outcomes, along with the duration of CRRT, RRT, ICU stay and hospitalization, which they did not report. Recently, a study published in 2015, in which 32,045 patients were stratified between July 2000 and October 2008 according to the KDIGO definition and classification of AKI, reported that patients who met both the serum creatinine level and urine output criteria for AKI were at the greatest risk of death or RRT [48]. However, the researchers investigated the outcomes of patients at various stages of AKI (only approximately 30 % of the patients required RRT) and of those without AKI with a mean age below 60 years. In contrast, we investigated the outcomes of critically ill, elderly patients with a median age of approximately 75 years, who had severe AKI and required CRRT.

In this study, we used the body-weight-adjusted median 6-h urine output immediately before CRRT initiation as the criterion for CRRT initiation. The early CRRT group had significantly better survival than the late CRRT group, even after multivariate analyses of risk factors. We chose the quantity of urine as a threshold for CRRT initiation for several reasons. First, urine output has long been used as a marker of AKI and for guiding renal resuscitation in critically ill patients [49–52]. In several previous studies urine output was also used as an indication to start CRRT, with reported survival benefit consistent with ours [33, 34, 46, 47]. Second, researchers have questioned whether BUN is an appropriate biomarker for determining when to initiate dialysis [36]. Urine output markers other than BUN have been reported to be potentially more suitable for prediction of the survival of critically ill patients with AKI [31, 47]. One RCT conducted in 1986 reported that urine output was a significant factor for survival. However, this study did nto demonstrate the utility of BUN as an indicator for starting dialysis [31]. A study performed in 2013 that compared BUN and urine output as criteria for the timing of CRRT initiation in critically ill patients with AKI concluded that urine quantity, but not the BUN concentration, was significantly associated with prognosis [47]. Third, although some novel biomarkers have been recently introduced as potentially better predictors of AKI, their applicability as markers of severity remain limited in clinical practice, especially in emergency departments and ICUs.

Numerous studies have explored the utilization of early biomarkers of AKI, such as NGAL and the recent combination of tissue inhibitor of metalloproteinase-2 and urine insulin-like growth factor-binding protein 7, in critically ill patients during the last decade [53–59]. However, for critically ill patients with AKI, biomarkers that reflect their ever-changing states are needed, and there is limited time to wait for test results. Urine output can be monitored very easily and allows for continuous measurements. Therefore, it could be used as a general tool for managing patients with AKI, including initial screening, risk assessment, diagnosis, treatment decisions, and assessment of prognosis. In our study, the time from ICU admission to CRRT initiation did not significantly differ between the groups (6.8 (1.5–31.1) hours in the early CRRT group versus 7.9 (1.7–37.8) hours in the late CRRT group, *P* = 0.30). This non-significant difference is in conflict with recent RCTs demonstrating significant differences in the time from ICU admission to RRT initiation [37, 38].

These discrepant results between our study and previous studies may be attributed to the use of different definitions of CRRT onset time [37, 38], in addition to differences in clinical practice policies. A trend toward admitting patients to the ICU at earlier time points could have decreased the difference in the time between CRRT initiation and ICU admission between the groups. A previous study conducted at our institute demonstrated that the time from ICU admission to CRRT initiation did not significantly differ between patients with high and low urine output at CRRT initiation, supporting the notion that institutional policy characteristics may be partly responsible for our non-significant findings [47].

To the best of our knowledge, this is the largest study to show a survival benefit of early CRRT initiation in critically ill, elderly patients with AKI. Our results were obtained from a multicenter, prospective observational study. Despite the increasing emphasis on the importance of care for the elderly population and increase in general medical management as care evolves, few studies have specifically examined CRRT in older individuals. Our data justify starting CRRT earlier in the course of severe AKI in elderly patients. Although the worse outcomes in the late CRRT group can be expected considering the univariate analysis results because of their lower blood pressure and higher PT-INR, total bilirubin, AST and ALT, the survival benefit of early CRRT initiation remained, even after multivariate risk factor analyses using the multivariate Cox regression model and PSM with adjustments for significant factors for mortality. We showed a significant survival benefit of early initiation of CRRT in the elderly before and after PSM (Figs. 1 and 2) and also demonstrated survival benefit in multivariate analyses with adjustments for various factors that influence the survival of patients with AKI (Table 2; model 3: HR 1.35, 95 % CI 1.06, 1.71, *P* = 0.02). The adjusted factors that influence survival in univariate analyses included age, sex, the CCI, SBP, prothrombin time, the total bilirubin, AST, and ALT levels, cumulative fluid balance, and use of diuretics. The significance of these results was maintained even after comparison with the propensity score-matched control group (Table 2; model 4: HR 1.32, 95 % CI 1.08, 1.61, *P* < 0.01). Additionally, on subgroup analysis of survivors, the total duration of hospitalization from CRRT initiation was shorter when CRRT was started earlier in the course of AKI (Table 3; 26.7 (16.1–38.2) versus 39.1 (27.7–79.3) days, *P* = 0.04). Although the duration of CRRT, total RRT, and the ICU stay from the start of CRRT did not differ based on the timing of CRRT initiation, our data may be used to guide clinicians in making difficult decisions about the management of critically ill, elderly patients with AKI.

Our study had several limitations. First, even after adjusting for multiple confounders and selection effects, subjects who possibly did not require CRRT may have been included in the early initiation group. Second, the quantity of urine is also an imperfect surrogate for renal function. It is affected by not only glomerular filtration but also volume status, urinary tract obstruction, and pharmacological therapy. However, our aim was not to find an indicator of kidney function but rather to identify a clinically useful marker that could predict the survival of patients with AKI. Third, our data are limited by the definition of the timing of CRRT initiation. In this study, urine output was the only variable that divided the study population in terms of the timing of dialysis initiation; several previous studies have used serum creatinine [60] or BUN [31, 36, 47] as a marker along with urine output. However, as stated previously, the use of BUN has been challenged in many recent studies investigating the association between the timing of dialysis initiation and mortality [36, 47], and to the best of our knowledge, no study has assessed the superiority of serum creatinine over urine output as a marker for determining the timing of dialysis initiation. Fourth, because the arterial partial pressure of oxygen was not known for the participants in this cohort, we were unable to determine the ratio of the arterial oxygen concentration to the fraction of inspired oxygen.

## Conclusions

In conclusion, a better prognosis can be expected if CRRT is applied early in the course of AKI in critically ill, elderly patients. Although well-designed RCTs are still necessary, our results contribute to the determination of whether early CRRT initiation yields a better survival benefit in elderly patients with severe AKI.

## Abbreviations

AKI, acute kidney injury; ALT, alanine aminotransferase; APACHE II score, acute physiology and chronic health evaluation II score; AST, aspartate aminotransferase; BUN, blood urea nitrogen; CCI, Charlson comorbidity index; CI, confidence interval; CRRT, continuous renal replacement therapy; eGFR, estimated glomerular filtration rate; HR, hazard ratio; ICU, intensive care unit; KDIGO, Kidney Disease: Improving Global Outcomes; PSM, propensity score matching; PT-INR, prothrombin time-international normalized ratio; RCT, randomized controlled trial; SBP, systolic blood pressure; SOFA score, sequential organ failure assessment score

## Additional file

Additional file 1: Figure S1. Distribution of propensity scores of the patients before and after propensity score matching. The propensity scores of the unmatched patients were significantly different between the early and late CRRT groups. The propensity scores of the matched patients were nearly identical between the two groups. (TIF 1300 kb)
