# Supplementary figures and images for: Early initiation of continuous renal replacement therapy improves survival of elderly patients with acute kidney injury: a multicenter prospective cohort study
Source: Crit Care. 2016 Aug 16;20:260. doi: 10.1186/s13054-016-1437-8 (PMC4986348; doi:10.1186/s13054-016-1437-8)

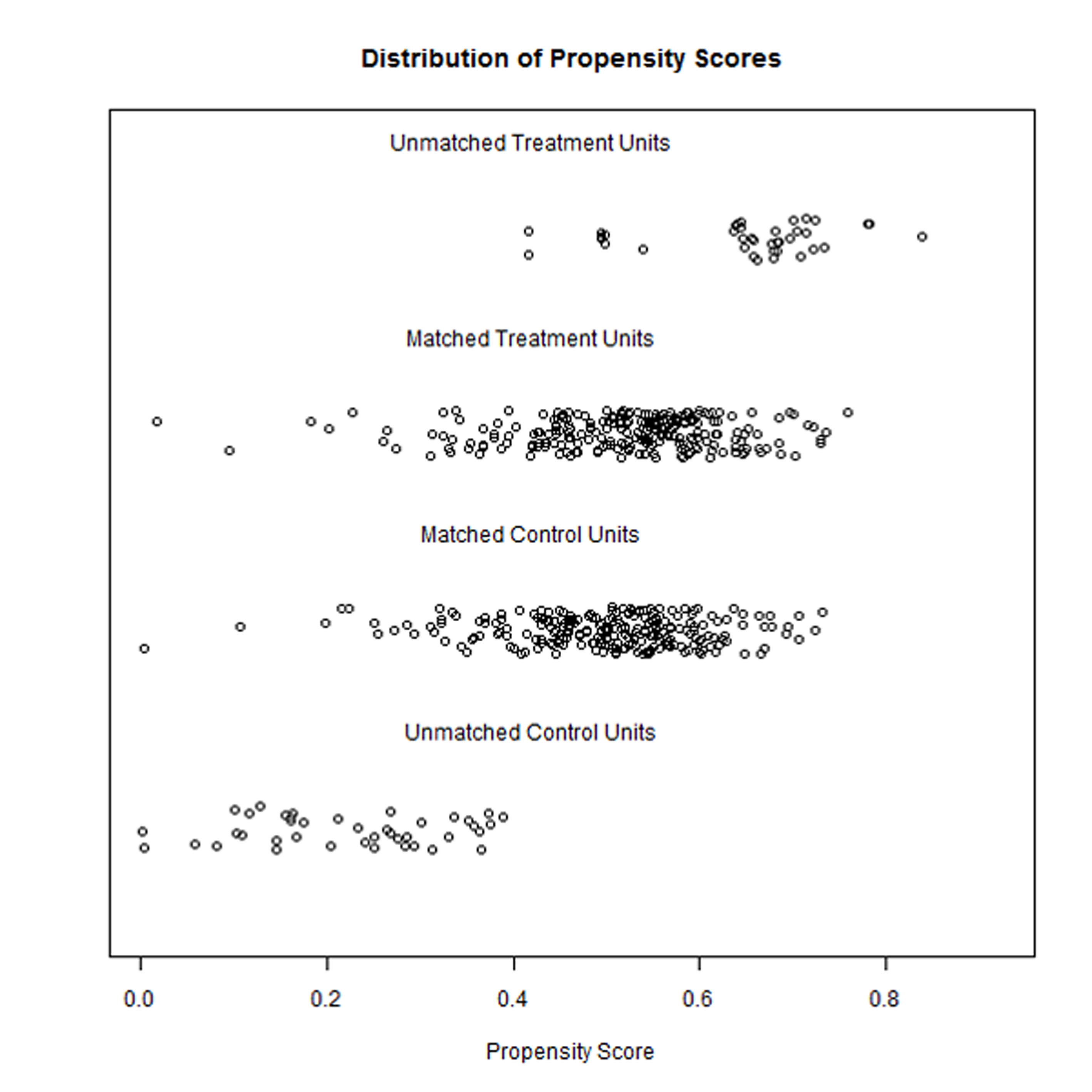

Supplement: Additional file 1: Figure S1. — Distribution of propensity scores of the patients before and after propensity score matching. The propensity scores of the unmatched patients were significantly different between the early and late CRRT groups. The propensity scores of the matched patients were nearly identical between the two groups. (TIF 1300 kb) [file 13054_2016_1437_MOESM1_ESM.tif]
